# Supplementary material for: A fault-intrusion-tolerant system and deadline-aware algorithm for scheduling scientific workflow in the cloud
Source: PeerJ Comput Sci. 2021 Nov 2;7:e747. doi: 10.7717/peerj-cs.747 (PMC8576554; doi:10.7717/peerj-cs.747)
Supplement: Supplemental Information 6 [file peerj-cs-07-747-s006.zip › Myproject1.1/docs/index-files/index-15.html]

P-Index


JavaScript is disabled on your browser.


- Overview
- Package
- Class
- Use
- Tree
- Deprecated
- Index
- Help

- Prev Letter
- Next Letter

- Frames
- No Frames

- All Classes

A B C D E F G H I J L M N O P Q R S T U V W Z 


## P

Packet - Interface in org.cloudbus.cloudsim
:   This class contains the structure for a network packet.

packetrecieved - Variable in class org.cloudbus.cloudsim.network.datacenter.NetworkHost


packetTohost - Variable in class org.cloudbus.cloudsim.network.datacenter.Switch


packetTosendGlobal - Variable in class org.cloudbus.cloudsim.network.datacenter.NetworkHost


packetTosendLocal - Variable in class org.cloudbus.cloudsim.network.datacenter.NetworkHost


ParameterException - Exception in org.cloudbus.cloudsim
:   This exception is to report bad or invalid parameters given during constructor.

ParameterException() - Constructor for exception org.cloudbus.cloudsim.ParameterException
:   Constructs a new exception with null as its detail message.

ParameterException(String) - Constructor for exception org.cloudbus.cloudsim.ParameterException
:   Creates a new ParameterException object.

Parameters - Class in org.workflowsim.utils
:   This class includes most parameters a user can specify in a configuration
    file

Parameters() - Constructor for class org.workflowsim.utils.Parameters


Parameters.FTCFailure - Enum in org.workflowsim.utils


Parameters.FTCMethod - Enum in org.workflowsim.utils
:   Fault Tolerant Clustering method

Parameters.FTCMonitor - Enum in org.workflowsim.utils


Parameters.PLNMethod - Enum in org.workflowsim.utils
:   Planning Algorithm (Global Scheduling Algorithm)

Parameters.SCHMethod - Enum in org.workflowsim.utils


ParetoDistr - Class in org.cloudbus.cloudsim.distributions
:   The Class ParetoDistr.

ParetoDistr(Random, double, double) - Constructor for class org.cloudbus.cloudsim.distributions.ParetoDistr
:   Instantiates a new pareto distr.

ParetoDistr(double, double) - Constructor for class org.cloudbus.cloudsim.distributions.ParetoDistr
:   Instantiates a new pareto distr.

parse() - Method in class org.workflowsim.WorkflowParser
:   Start to parse a workflow which includes text files and xml files.

parseExperimentName(String) - Static method in class org.cloudbus.cloudsim.examples.power.Helper
:   Parses the experiment name.

parser - Variable in class org.workflowsim.WorkflowPlanner
:   The workflow parser.

pause(int, double) - Static method in class org.cloudbus.cloudsim.core.CloudSim
:   Used to pause an entity for some time.

pause(double) - Method in class org.cloudbus.cloudsim.core.SimEntity
:   Set the entity to be inactive for a time period.

PAUSED - Static variable in class org.cloudbus.cloudsim.Cloudlet
:   The Cloudlet has been paused.

pauseSimulation() - Static method in class org.cloudbus.cloudsim.core.CloudSim
:   This method is called if one wants to pause the simulation.

pauseSimulation(long) - Static method in class org.cloudbus.cloudsim.core.CloudSim
:   This method is called if one wants to pause the simulation at a given time.

Pe - Class in org.cloudbus.cloudsim
:   CloudSim Pe (Processing Element) class represents CPU unit, defined in terms of Millions
    Instructions Per Second (MIPS) rating.  
    **ASSUMPTION: **All PEs under the same Machine have the same MIPS rating.****

Pe(int, PeProvisioner) - Constructor for class org.cloudbus.cloudsim.Pe
:   Allocates a new Pe object.

PeList - Class in org.cloudbus.cloudsim.lists
:   PeList is a collection of operations on lists of PEs.

PeList() - Constructor for class org.cloudbus.cloudsim.lists.PeList


PeProvisioner - Class in org.cloudbus.cloudsim.provisioners
:   The Class PeProvisioner.

PeProvisioner(double) - Constructor for class org.cloudbus.cloudsim.provisioners.PeProvisioner
:   Creates the new PeProvisioner.

PeProvisionerSimple - Class in org.cloudbus.cloudsim.provisioners
:   The Class PeProvisionerSimple.

PeProvisionerSimple(double) - Constructor for class org.cloudbus.cloudsim.provisioners.PeProvisionerSimple
:   Creates the PeProvisionerSimple object.

PES\_NUMBER - Static variable in class org.cloudbus.cloudsim.network.datacenter.NetworkConstants


PipelineRuntimeVariance - Class in org.workflowsim.clustering.balancing.metrics
:   The PipelineRuntimeVariance is the standard deviation of the runtime of the pipelines

PipelineRuntimeVariance() - Constructor for class org.workflowsim.clustering.balancing.metrics.PipelineRuntimeVariance


PKT\_SIZE - Static variable in class org.cloudbus.cloudsim.DataCloudTags
:   The default packet size (in byte) for sending events to other entity.

pktlist - Variable in class org.cloudbus.cloudsim.network.datacenter.Switch


pktrecv - Variable in class org.cloudbus.cloudsim.network.datacenter.NetworkCloudletSpaceSharedScheduler


pkttosend - Variable in class org.cloudbus.cloudsim.network.datacenter.NetworkCloudletSpaceSharedScheduler


PlanetLabConstants - Class in org.cloudbus.cloudsim.examples.power.planetlab
:   If you are using any algorithms, policies or workload included in the power package please cite
    the following paper:
    Anton Beloglazov, and Rajkumar Buyya, "Optimal Online Deterministic Algorithms and Adaptive
    Heuristics for Energy and Performance Efficient Dynamic Consolidation of Virtual Machines in
    Cloud Data Centers", Concurrency and Computation: Practice and Experience (CCPE), Volume 24,
    Issue 13, Pages: 1397-1420, John Wiley & Sons, Ltd, New York, USA, 2012

PlanetLabConstants() - Constructor for class org.cloudbus.cloudsim.examples.power.planetlab.PlanetLabConstants


PlanetLabHelper - Class in org.cloudbus.cloudsim.examples.power.planetlab
:   A helper class for the running examples for the PlanetLab workload.

PlanetLabHelper() - Constructor for class org.cloudbus.cloudsim.examples.power.planetlab.PlanetLabHelper


PlanetLabRunner - Class in org.cloudbus.cloudsim.examples.power.planetlab
:   The example runner for the PlanetLab workload.

PlanetLabRunner(boolean, boolean, String, String, String, String, String, String) - Constructor for class org.cloudbus.cloudsim.examples.power.planetlab.PlanetLabRunner
:   Instantiates a new planet lab runner.

PlanetLabRunnerConsole - Class in org.cloudbus.cloudsim.examples.power.planetlab
:   This is a universal example runner that can be used for running examples from console.

PlanetLabRunnerConsole() - Constructor for class org.cloudbus.cloudsim.examples.power.planetlab.PlanetLabRunnerConsole


PlannerInterface - Interface in org.workflowsim.planning
:   The Planner interface

postFailureRecord(FailureRecord) - Static method in class org.workflowsim.failure.FailureMonitor
:   A post from a broker so that we can update record list

PowerDatacenter - Class in org.cloudbus.cloudsim.power
:   PowerDatacenter is a class that enables simulation of power-aware data centers.

PowerDatacenter(String, DatacenterCharacteristics, VmAllocationPolicy, List<Storage>, double) - Constructor for class org.cloudbus.cloudsim.power.PowerDatacenter
:   Instantiates a new datacenter.

PowerDatacenterBroker - Class in org.cloudbus.cloudsim.power
:   A broker for the power package.

PowerDatacenterBroker(String) - Constructor for class org.cloudbus.cloudsim.power.PowerDatacenterBroker
:   Instantiates a new power datacenter broker.

PowerDatacenterNonPowerAware - Class in org.cloudbus.cloudsim.power
:   PowerDatacenterNonPowerAware is a class that represents a non-power aware data center in the
    context of power-aware simulations.

PowerDatacenterNonPowerAware(String, DatacenterCharacteristics, VmAllocationPolicy, List<Storage>, double) - Constructor for class org.cloudbus.cloudsim.power.PowerDatacenterNonPowerAware
:   Instantiates a new datacenter.

PowerHost - Class in org.cloudbus.cloudsim.power
:   PowerHost class enables simulation of power-aware hosts.

PowerHost(int, RamProvisioner, BwProvisioner, long, List<? extends Pe>, VmScheduler, PowerModel) - Constructor for class org.cloudbus.cloudsim.power.PowerHost
:   Instantiates a new host.

PowerHostUtilizationHistory - Class in org.cloudbus.cloudsim.power
:   The class of a host that stores its CPU utilization history.

PowerHostUtilizationHistory(int, RamProvisioner, BwProvisioner, long, List<? extends Pe>, VmScheduler, PowerModel) - Constructor for class org.cloudbus.cloudsim.power.PowerHostUtilizationHistory
:   Instantiates a new power host utilization history.

PowerModel - Interface in org.cloudbus.cloudsim.power.models
:   The PowerModel interface needs to be implemented in order to provide a model of power consumption
    depending on utilization for system components.

PowerModelCubic - Class in org.cloudbus.cloudsim.power.models
:   The Class PowerModelCubic.

PowerModelCubic(double, double) - Constructor for class org.cloudbus.cloudsim.power.models.PowerModelCubic
:   Instantiates a new power model cubic.

PowerModelLinear - Class in org.cloudbus.cloudsim.power.models
:   The Class PowerModelLinear.

PowerModelLinear(double, double) - Constructor for class org.cloudbus.cloudsim.power.models.PowerModelLinear
:   Instantiates a new linear power model.

PowerModelSpecPower - Class in org.cloudbus.cloudsim.power.models
:   The abstract class of power models created based on data from SPECpower benchmark:
    http://www.spec.org/power\_ssj2008/
    If you are using any algorithms, policies or workload included in the power package, please cite
    the following paper:
    Anton Beloglazov, and Rajkumar Buyya, "Optimal Online Deterministic Algorithms and Adaptive
    Heuristics for Energy and Performance Efficient Dynamic Consolidation of Virtual Machines in
    Cloud Data Centers", Concurrency and Computation: Practice and Experience (CCPE), Volume 24,
    Issue 13, Pages: 1397-1420, John Wiley & Sons, Ltd, New York, USA, 2012

PowerModelSpecPower() - Constructor for class org.cloudbus.cloudsim.power.models.PowerModelSpecPower


PowerModelSpecPowerHpProLiantMl110G3PentiumD930 - Class in org.cloudbus.cloudsim.power.models
:   The power model of an HP ProLiant ML110 G3 (1 x [Pentium D930 3000 MHz, 2 cores], 4GB).

PowerModelSpecPowerHpProLiantMl110G3PentiumD930() - Constructor for class org.cloudbus.cloudsim.power.models.PowerModelSpecPowerHpProLiantMl110G3PentiumD930


PowerModelSpecPowerHpProLiantMl110G4Xeon3040 - Class in org.cloudbus.cloudsim.power.models
:   The power model of an HP ProLiant ML110 G4 (1 x [Xeon 3040 1860 MHz, 2 cores], 4GB).

PowerModelSpecPowerHpProLiantMl110G4Xeon3040() - Constructor for class org.cloudbus.cloudsim.power.models.PowerModelSpecPowerHpProLiantMl110G4Xeon3040


PowerModelSpecPowerHpProLiantMl110G5Xeon3075 - Class in org.cloudbus.cloudsim.power.models
:   The power model of an HP ProLiant ML110 G5 (1 x [Xeon 3075 2660 MHz, 2 cores], 4GB).

PowerModelSpecPowerHpProLiantMl110G5Xeon3075() - Constructor for class org.cloudbus.cloudsim.power.models.PowerModelSpecPowerHpProLiantMl110G5Xeon3075


PowerModelSpecPowerIbmX3250XeonX3470 - Class in org.cloudbus.cloudsim.power.models
:   The power model of an IBM server x3250 (1 x [Xeon X3470 2933 MHz, 4 cores], 8GB).

PowerModelSpecPowerIbmX3250XeonX3470() - Constructor for class org.cloudbus.cloudsim.power.models.PowerModelSpecPowerIbmX3250XeonX3470


PowerModelSpecPowerIbmX3250XeonX3480 - Class in org.cloudbus.cloudsim.power.models
:   The power model of an IBM server x3250 (1 x [Xeon X3480 3067 MHz, 4 cores], 8GB).

PowerModelSpecPowerIbmX3250XeonX3480() - Constructor for class org.cloudbus.cloudsim.power.models.PowerModelSpecPowerIbmX3250XeonX3480


PowerModelSpecPowerIbmX3550XeonX5670 - Class in org.cloudbus.cloudsim.power.models
:   The power model of an IBM server x3550 (2 x [Xeon X5670 2933 MHz, 6 cores], 12GB).

PowerModelSpecPowerIbmX3550XeonX5670() - Constructor for class org.cloudbus.cloudsim.power.models.PowerModelSpecPowerIbmX3550XeonX5670


PowerModelSpecPowerIbmX3550XeonX5675 - Class in org.cloudbus.cloudsim.power.models
:   The power model of an IBM server x3550 (2 x [Xeon X5675 3067 MHz, 6 cores], 16GB).

PowerModelSpecPowerIbmX3550XeonX5675() - Constructor for class org.cloudbus.cloudsim.power.models.PowerModelSpecPowerIbmX3550XeonX5675


PowerModelSqrt - Class in org.cloudbus.cloudsim.power.models
:   The Class PowerModelSqrt.

PowerModelSqrt(double, double) - Constructor for class org.cloudbus.cloudsim.power.models.PowerModelSqrt
:   Instantiates a new power model sqrt.

PowerModelSquare - Class in org.cloudbus.cloudsim.power.models
:   The Class PowerModelSquare.

PowerModelSquare(double, double) - Constructor for class org.cloudbus.cloudsim.power.models.PowerModelSquare
:   Instantiates a new power model square.

PowerVm - Class in org.cloudbus.cloudsim.power
:   The class of a VM that stores its CPU utilization history.

PowerVm(int, int, double, int, int, long, long, int, String, CloudletScheduler, double) - Constructor for class org.cloudbus.cloudsim.power.PowerVm
:   Instantiates a new power vm.

PowerVmAllocationPolicyAbstract - Class in org.cloudbus.cloudsim.power
:   The class of an abstract power-aware VM allocation policy.

PowerVmAllocationPolicyAbstract(List<? extends Host>) - Constructor for class org.cloudbus.cloudsim.power.PowerVmAllocationPolicyAbstract
:   Instantiates a new power vm allocation policy abstract.

PowerVmAllocationPolicyMigrationAbstract - Class in org.cloudbus.cloudsim.power
:   The class of an abstract power-aware VM allocation policy that dynamically optimizes the VM
    allocation using migration.

PowerVmAllocationPolicyMigrationAbstract(List<? extends Host>, PowerVmSelectionPolicy) - Constructor for class org.cloudbus.cloudsim.power.PowerVmAllocationPolicyMigrationAbstract
:   Instantiates a new power vm allocation policy migration abstract.

PowerVmAllocationPolicyMigrationInterQuartileRange - Class in org.cloudbus.cloudsim.power
:   The Inter Quartile Range (IQR) VM allocation policy.

PowerVmAllocationPolicyMigrationInterQuartileRange(List<? extends Host>, PowerVmSelectionPolicy, double, PowerVmAllocationPolicyMigrationAbstract, double) - Constructor for class org.cloudbus.cloudsim.power.PowerVmAllocationPolicyMigrationInterQuartileRange
:   Instantiates a new power vm allocation policy migration mad.

PowerVmAllocationPolicyMigrationInterQuartileRange(List<? extends Host>, PowerVmSelectionPolicy, double, PowerVmAllocationPolicyMigrationAbstract) - Constructor for class org.cloudbus.cloudsim.power.PowerVmAllocationPolicyMigrationInterQuartileRange
:   Instantiates a new power vm allocation policy migration mad.

PowerVmAllocationPolicyMigrationLocalRegression - Class in org.cloudbus.cloudsim.power
:   The Local Regression (LR) VM allocation policy.

PowerVmAllocationPolicyMigrationLocalRegression(List<? extends Host>, PowerVmSelectionPolicy, double, double, PowerVmAllocationPolicyMigrationAbstract, double) - Constructor for class org.cloudbus.cloudsim.power.PowerVmAllocationPolicyMigrationLocalRegression
:   Instantiates a new power vm allocation policy migration local regression.

PowerVmAllocationPolicyMigrationLocalRegression(List<? extends Host>, PowerVmSelectionPolicy, double, double, PowerVmAllocationPolicyMigrationAbstract) - Constructor for class org.cloudbus.cloudsim.power.PowerVmAllocationPolicyMigrationLocalRegression
:   Instantiates a new power vm allocation policy migration local regression.

PowerVmAllocationPolicyMigrationLocalRegressionRobust - Class in org.cloudbus.cloudsim.power
:   The Local Regression Robust (LRR) VM allocation policy.

PowerVmAllocationPolicyMigrationLocalRegressionRobust(List<? extends Host>, PowerVmSelectionPolicy, double, double, PowerVmAllocationPolicyMigrationAbstract, double) - Constructor for class org.cloudbus.cloudsim.power.PowerVmAllocationPolicyMigrationLocalRegressionRobust
:   Instantiates a new power vm allocation policy migration local regression.

PowerVmAllocationPolicyMigrationLocalRegressionRobust(List<? extends Host>, PowerVmSelectionPolicy, double, double, PowerVmAllocationPolicyMigrationAbstract) - Constructor for class org.cloudbus.cloudsim.power.PowerVmAllocationPolicyMigrationLocalRegressionRobust
:   Instantiates a new power vm allocation policy migration local regression.

PowerVmAllocationPolicyMigrationMedianAbsoluteDeviation - Class in org.cloudbus.cloudsim.power
:   The Median Absolute Deviation (MAD) VM allocation policy.

PowerVmAllocationPolicyMigrationMedianAbsoluteDeviation(List<? extends Host>, PowerVmSelectionPolicy, double, PowerVmAllocationPolicyMigrationAbstract, double) - Constructor for class org.cloudbus.cloudsim.power.PowerVmAllocationPolicyMigrationMedianAbsoluteDeviation
:   Instantiates a new power vm allocation policy migration mad.

PowerVmAllocationPolicyMigrationMedianAbsoluteDeviation(List<? extends Host>, PowerVmSelectionPolicy, double, PowerVmAllocationPolicyMigrationAbstract) - Constructor for class org.cloudbus.cloudsim.power.PowerVmAllocationPolicyMigrationMedianAbsoluteDeviation
:   Instantiates a new power vm allocation policy migration mad.

PowerVmAllocationPolicyMigrationStaticThreshold - Class in org.cloudbus.cloudsim.power
:   The Static Threshold (THR) VM allocation policy.

PowerVmAllocationPolicyMigrationStaticThreshold(List<? extends Host>, PowerVmSelectionPolicy, double) - Constructor for class org.cloudbus.cloudsim.power.PowerVmAllocationPolicyMigrationStaticThreshold
:   Instantiates a new power vm allocation policy migration mad.

PowerVmAllocationPolicySimple - Class in org.cloudbus.cloudsim.power
:   This a simple class representing a simple VM allocation policy that does not perform any
    optimization of the VM allocation.

PowerVmAllocationPolicySimple(List<? extends Host>) - Constructor for class org.cloudbus.cloudsim.power.PowerVmAllocationPolicySimple
:   Instantiates a new power vm allocation policy simple.

PowerVmList - Class in org.cloudbus.cloudsim.power.lists
:   PowerVmList is a collection of operations on lists of power-enabled VMs.

PowerVmList() - Constructor for class org.cloudbus.cloudsim.power.lists.PowerVmList


PowerVmSelectionPolicy - Class in org.cloudbus.cloudsim.power
:   The class of an abstract VM selection policy.

PowerVmSelectionPolicy() - Constructor for class org.cloudbus.cloudsim.power.PowerVmSelectionPolicy


PowerVmSelectionPolicyMaximumCorrelation - Class in org.cloudbus.cloudsim.power
:   The Maximum Correlation (MC) VM selection policy.

PowerVmSelectionPolicyMaximumCorrelation(PowerVmSelectionPolicy) - Constructor for class org.cloudbus.cloudsim.power.PowerVmSelectionPolicyMaximumCorrelation
:   Instantiates a new power vm selection policy maximum correlation.

PowerVmSelectionPolicyMinimumMigrationTime - Class in org.cloudbus.cloudsim.power
:   The Minimum Migration Time (MMT) VM selection policy.

PowerVmSelectionPolicyMinimumMigrationTime() - Constructor for class org.cloudbus.cloudsim.power.PowerVmSelectionPolicyMinimumMigrationTime


PowerVmSelectionPolicyMinimumUtilization - Class in org.cloudbus.cloudsim.power
:   The Minimum Utilization (MU) VM selection policy.

PowerVmSelectionPolicyMinimumUtilization() - Constructor for class org.cloudbus.cloudsim.power.PowerVmSelectionPolicyMinimumUtilization


PowerVmSelectionPolicyRandomSelection - Class in org.cloudbus.cloudsim.power
:   The Random Selection (RS) VM selection policy.

PowerVmSelectionPolicyRandomSelection() - Constructor for class org.cloudbus.cloudsim.power.PowerVmSelectionPolicyRandomSelection


Predicate - Class in org.cloudbus.cloudsim.core.predicates
:   Predicates are used to select events from the deferred queue.

Predicate() - Constructor for class org.cloudbus.cloudsim.core.predicates.Predicate


PredicateAny - Class in org.cloudbus.cloudsim.core.predicates
:   A predicate which will match any event on the deferred event queue.

PredicateAny() - Constructor for class org.cloudbus.cloudsim.core.predicates.PredicateAny


PredicateFrom - Class in org.cloudbus.cloudsim.core.predicates
:   A predicate which selects events from specific entities.  
    The idea of simulation predicates was copied from SimJava 2.

PredicateFrom(int) - Constructor for class org.cloudbus.cloudsim.core.predicates.PredicateFrom
:   Constructor used to select events that were sent by a specific entity.

PredicateFrom(int[]) - Constructor for class org.cloudbus.cloudsim.core.predicates.PredicateFrom
:   Constructor used to select events that were sent by any entity from a given set.

PredicateNone - Class in org.cloudbus.cloudsim.core.predicates
:   A predicate which will **not** match any event on the deferred event queue.

PredicateNone() - Constructor for class org.cloudbus.cloudsim.core.predicates.PredicateNone


PredicateNotFrom - Class in org.cloudbus.cloudsim.core.predicates
:   A predicate which selects events that have not been sent by specific entities.

PredicateNotFrom(int) - Constructor for class org.cloudbus.cloudsim.core.predicates.PredicateNotFrom
:   Constructor used to select events that were not sent by a specific entity.

PredicateNotFrom(int[]) - Constructor for class org.cloudbus.cloudsim.core.predicates.PredicateNotFrom
:   Constructor used to select events that were not sent by any entity from a given set.

PredicateNotType - Class in org.cloudbus.cloudsim.core.predicates
:   A predicate to select events that don't match specific tags.

PredicateNotType(int) - Constructor for class org.cloudbus.cloudsim.core.predicates.PredicateNotType
:   Constructor used to select events whose tags do not match a given tag.

PredicateNotType(int[]) - Constructor for class org.cloudbus.cloudsim.core.predicates.PredicateNotType
:   Constructor used to select events whose tag values do not match any of the given tags.

PredicateType - Class in org.cloudbus.cloudsim.core.predicates
:   A predicate to select events with specific tags.

PredicateType(int) - Constructor for class org.cloudbus.cloudsim.core.predicates.PredicateType
:   Constructor used to select events with the tag value `t1`.

PredicateType(int[]) - Constructor for class org.cloudbus.cloudsim.core.predicates.PredicateType
:   Constructor used to select events with a tag value equal to any of the specified tags.

predictFileTransferTime(List<String>) - Method in class org.cloudbus.cloudsim.Datacenter
:   Predict file transfer time.

print(String) - Static method in class org.cloudbus.cloudsim.Log
:   Prints the message.

print(Object) - Static method in class org.cloudbus.cloudsim.Log
:   Prints the message passed as a non-String object.

printCloudletList(List<Cloudlet>) - Static method in class org.cloudbus.cloudsim.examples.power.Helper
:   Prints the Cloudlet objects.

printLine(String) - Static method in class org.cloudbus.cloudsim.Log
:   Prints the line.

printLine() - Static method in class org.cloudbus.cloudsim.Log
:   Prints the empty line.

printLine(Object) - Static method in class org.cloudbus.cloudsim.Log
:   Prints the line passed as a non-String object.

printMetricHistory(List<? extends Host>, PowerVmAllocationPolicyMigrationAbstract) - Static method in class org.cloudbus.cloudsim.examples.power.Helper
:   Prints the metric history.

printMetrics() - Method in class org.workflowsim.clustering.balancing.BalancedClustering
:   Print out all the balancing metrics

printOverUtilizedHosts(List<PowerHostUtilizationHistory>) - Method in class org.cloudbus.cloudsim.power.PowerVmAllocationPolicyMigrationAbstract
:   Prints the over utilized hosts.

printResults(PowerDatacenter, List<Vm>, double, String, boolean, String) - Static method in class org.cloudbus.cloudsim.examples.power.Helper
:   Prints the results.

printVersion() - Method in class org.workflowsim.utils.ArgumentParser


printVersion() - Static method in class org.workflowsim.utils.Parameters


process(ArrayList<TaskSet>) - Method in class org.workflowsim.clustering.balancing.methods.HorizontalDistanceBalancing
:   Sort taskSet based on their impact factors and then merge similar taskSet together

process(ArrayList<TaskSet>) - Method in class org.workflowsim.clustering.balancing.methods.HorizontalImpactBalancing
:   Sort taskSet based on their impact factors and then merge similar taskSet together

process(Job, int) - Static method in class org.workflowsim.reclustering.ReclusteringEngine
:   Process job recreation based on different reclustering algorithm

processCloudlet(SimEvent, int) - Method in class org.cloudbus.cloudsim.Datacenter
:   Processes a Cloudlet based on the event type.

processCloudletCancel(int, int, int) - Method in class org.cloudbus.cloudsim.Datacenter
:   Processes a Cloudlet cancel request.

processCloudletCheck(SimEvent) - Method in class org.workflowsim.WorkflowScheduler
:   process cloudlet (job) check (not supported yet)

processCloudletMove(int[], int) - Method in class org.cloudbus.cloudsim.Datacenter
:   Process the event for an User/Broker who wants to move a Cloudlet.

processCloudletPause(int, int, int, boolean) - Method in class org.cloudbus.cloudsim.Datacenter
:   Processes a Cloudlet pause request.

processCloudletResume(int, int, int, boolean) - Method in class org.cloudbus.cloudsim.Datacenter
:   Processes a Cloudlet resume request.

processCloudletReturn(SimEvent) - Method in class org.cloudbus.cloudsim.DatacenterBroker
:   Process a cloudlet return event.

processCloudletReturn(SimEvent) - Method in class org.cloudbus.cloudsim.network.datacenter.NetDatacenterBroker
:   Process a cloudlet return event.

processCloudletReturn(SimEvent) - Method in class org.workflowsim.WorkflowScheduler
:   Process a cloudlet (job) return event.

processCloudletStatus(SimEvent) - Method in class org.cloudbus.cloudsim.Datacenter
:   Process the event for an User/Broker who wants to know the status of a Cloudlet.

processCloudletSubmit(SimEvent, boolean) - Method in class org.cloudbus.cloudsim.Datacenter
:   Processes a Cloudlet submission.

processCloudletSubmit(SimEvent, boolean) - Method in class org.cloudbus.cloudsim.network.datacenter.NetworkDatacenter
:   Processes a Cloudlet submission.

processCloudletSubmit(SimEvent, boolean) - Method in class org.cloudbus.cloudsim.power.PowerDatacenter


processCloudletSubmit(SimEvent, boolean) - Method in class org.workflowsim.DatacenterExtended
:   Processes a Cloudlet submission.

processCloudletSubmit(SimEvent) - Method in class org.workflowsim.WorkflowScheduler
:   Submits cloudlet (job) list

processCloudletUpdate(SimEvent) - Method in class org.workflowsim.WorkflowScheduler
:   Update a cloudlet (job)

processClustering() - Method in class org.workflowsim.ClusteringEngine
:   Processes events available for this ClusteringEngine.

processDataAdd(SimEvent, boolean) - Method in class org.cloudbus.cloudsim.Datacenter
:   Process data add.

processDataDelete(SimEvent, boolean) - Method in class org.cloudbus.cloudsim.Datacenter
:   Process data del.

processDataStageIn(List<File>, Cloudlet) - Method in class org.workflowsim.DatacenterExtended


processDatastaging() - Method in class org.workflowsim.ClusteringEngine
:   Adds data stage-in jobs to the job list

processEndSimulation() - Method in class org.cloudbus.cloudsim.core.CloudInformationService
:   Notifies the registered entities about the end of simulation.

processEvent(SimEvent) - Method in class org.cloudbus.cloudsim.core.CloudInformationService
:   Processes events scheduled for this entity.

processEvent(SimEvent) - Method in class org.cloudbus.cloudsim.core.CloudSimShutdown
:   The main method that shuts down hostList and Cloud Information Service (GIS).

processEvent(SimEvent) - Method in class org.cloudbus.cloudsim.core.SimEntity
:   This method is invoked by the `Simulation` class whenever there is an event in the
    deferred queue, which needs to be processed by the entity.

processEvent(SimEvent) - Method in class org.cloudbus.cloudsim.Datacenter
:   Processes events or services that are available for this PowerDatacenter.

processEvent(SimEvent) - Method in class org.cloudbus.cloudsim.DatacenterBroker
:   Processes events available for this Broker.

processEvent(SimEvent) - Method in class org.cloudbus.cloudsim.examples.CloudSimExample8.GlobalBroker


processEvent(SimEvent) - Method in class org.cloudbus.cloudsim.network.datacenter.NetDatacenterBroker
:   Processes events available for this Broker.

processEvent(SimEvent) - Method in class org.cloudbus.cloudsim.network.datacenter.Switch


processEvent(SimEvent) - Method in class org.workflowsim.ClusteringEngine
:   Processes events available for this Broker.

processEvent(SimEvent) - Method in class org.workflowsim.WorkflowEngine
:   Processes events available for this Broker.

processEvent(SimEvent) - Method in class org.workflowsim.WorkflowPlanner
:   Processes events available for this Broker.

processEvent(SimEvent) - Method in class org.workflowsim.WorkflowScheduler
:   Process an event

processhostpacket(SimEvent) - Method in class org.cloudbus.cloudsim.network.datacenter.Switch


processJobReturn(SimEvent) - Method in class org.workflowsim.WorkflowEngine
:   Process a job return event.

processJobSubmit(SimEvent) - Method in class org.workflowsim.WorkflowEngine
:   Process a submit event

processOtherEvent(SimEvent) - Method in class org.cloudbus.cloudsim.core.CloudInformationService
:   This method needs to override by a child class for processing other events.

processOtherEvent(SimEvent) - Method in class org.cloudbus.cloudsim.Datacenter
:   Here all the method related to VM requests will be received and forwarded to the related
    method.

processOtherEvent(SimEvent) - Method in class org.cloudbus.cloudsim.DatacenterBroker
:   Overrides this method when making a new and different type of Broker.

processOtherEvent(SimEvent) - Method in class org.cloudbus.cloudsim.network.datacenter.NetDatacenterBroker
:   Overrides this method when making a new and different type of Broker.

processOtherEvent(SimEvent) - Method in class org.workflowsim.ClusteringEngine
:   Overrides this method when making a new and different type of Broker.

processOtherEvent(SimEvent) - Method in class org.workflowsim.DatacenterExtended


processOtherEvent(SimEvent) - Method in class org.workflowsim.WorkflowEngine
:   Overrides this method when making a new and different type of Broker.

processOtherEvent(SimEvent) - Method in class org.workflowsim.WorkflowPlanner
:   Overrides this method when making a new and different type of Broker.

processpacket(SimEvent) - Method in class org.cloudbus.cloudsim.network.datacenter.Switch


processpacket\_down(SimEvent) - Method in class org.cloudbus.cloudsim.network.datacenter.AggregateSwitch
:   Send Packet to switch connected through a downlink port

processpacket\_down(SimEvent) - Method in class org.cloudbus.cloudsim.network.datacenter.Switch


processpacket\_up(SimEvent) - Method in class org.cloudbus.cloudsim.network.datacenter.AggregateSwitch
:   Send Packet to switch connected through a uplink port

processpacket\_up(SimEvent) - Method in class org.cloudbus.cloudsim.network.datacenter.EdgeSwitch
:   Send Packet to switch connected through a uplink port

processpacket\_up(SimEvent) - Method in class org.cloudbus.cloudsim.network.datacenter.RootSwitch
:   Send Packet to switch connected through a downlink port

processpacket\_up(SimEvent) - Method in class org.cloudbus.cloudsim.network.datacenter.Switch


processpacketforward(SimEvent) - Method in class org.cloudbus.cloudsim.network.datacenter.EdgeSwitch
:   Send Packet to hosts connected to the switch

processpacketforward(SimEvent) - Method in class org.cloudbus.cloudsim.network.datacenter.Switch


processPingRequest(SimEvent) - Method in class org.cloudbus.cloudsim.Datacenter
:   Processes a ping request.

processResourceCharacteristics(SimEvent) - Method in class org.cloudbus.cloudsim.DatacenterBroker
:   Process the return of a request for the characteristics of a PowerDatacenter.

processResourceCharacteristics(SimEvent) - Method in class org.cloudbus.cloudsim.network.datacenter.NetDatacenterBroker
:   Process the return of a request for the characteristics of a PowerDatacenter.

processResourceCharacteristicsRequest(SimEvent) - Method in class org.cloudbus.cloudsim.DatacenterBroker
:   Process a request for the characteristics of a PowerDatacenter.

processResourceCharacteristicsRequest(SimEvent) - Method in class org.cloudbus.cloudsim.network.datacenter.NetDatacenterBroker
:   Process a request for the characteristics of a PowerDatacenter.

processResourceCharacteristicsRequest(SimEvent) - Method in class org.workflowsim.WorkflowEngine
:   Process a request for the characteristics of a PowerDatacenter.

processResourceCharacteristicsRequest(SimEvent) - Method in class org.workflowsim.WorkflowScheduler
:   Process a request for the characteristics of a PowerDatacenter.

processVmCreate(SimEvent, boolean) - Method in class org.cloudbus.cloudsim.Datacenter
:   Process the event for an User/Broker who wants to create a VM in this PowerDatacenter.

processVmCreate(SimEvent) - Method in class org.cloudbus.cloudsim.DatacenterBroker
:   Process the ack received due to a request for VM creation.

processVmCreate(SimEvent) - Method in class org.cloudbus.cloudsim.power.PowerDatacenterBroker


processVmCreate(SimEvent) - Method in class org.workflowsim.WorkflowScheduler
:   Process the ack received due to a request for VM creation.

processVmCreateNetwork(Vm) - Method in class org.cloudbus.cloudsim.network.datacenter.NetworkDatacenter
:   Create the VM within the NetworkDatacenter.

processVmDestroy(SimEvent, boolean) - Method in class org.cloudbus.cloudsim.Datacenter
:   Process the event for an User/Broker who wants to destroy a VM previously created in this
    PowerDatacenter.

processVmMigrate(SimEvent, boolean) - Method in class org.cloudbus.cloudsim.Datacenter
:   Process the event for an User/Broker who wants to migrate a VM.

processVmMigrate(SimEvent, boolean) - Method in class org.cloudbus.cloudsim.power.PowerDatacenter

A B C D E F G H I J L M N O P Q R S T U V W Z

- Overview
- Package
- Class
- Use
- Tree
- Deprecated
- Index
- Help

- Prev Letter
- Next Letter

- Frames
- No Frames

- All Classes
